# Glucocorticoid-induced osteoporosis: an update

**DOI:** 10.1007/s12020-018-1588-2

**Published:** 2018-04-24

**Authors:** Juliet Compston

**Affiliations:** Department of Medicine, Cambridge Biomedical Campus, Cambridge, CB2 0SL UK

**Keywords:** Glucocorticoids, Bone density, Fracture, Bisphosphonates, Teriparatide

## Abstract

Glucocorticoid-induced osteoporosis is the most common secondary cause of osteoporosis and the resulting fractures cause significant morbidity. Following initiation of oral glucocorticoids, rapid bone loss occurs, and fracture risk increases within a few months in a dose-dependent manner. These adverse effects are due to inhibition of bone formation accompanied by an early but transient increase in bone resorption. Multiple mechanisms underlie these changes in bone remodeling; direct effects include upregulation of PPARγR2, increased expression of sclerostin and increased RANKL/OPG ratio, whilst hypogonadism, altered renal and intestinal calcium handling, and reduced production of insulin-like growth factor 1 also contribute. Fracture risk assessment should be performed as soon as possible after glucocorticoids are initiated and bone protective therapy started promptly in individuals at high-risk, with calcium and vitamin D supplements where appropriate. Oral bisphosphonates are currently regarded as first line options on the grounds of their low cost. However, teriparatide has been shown to be superior in its effects on BMD and vertebral fracture risk in glucocorticoid-treated individuals with osteoporosis and should be considered as an alternative first line option in high-risk patients.

## Introduction

Glucocorticoids are used in the treatment of a wide range of diseases and it is estimated that 1–2% of the population is receiving long-term glucocorticoid therapy [1–4]. The adverse skeletal effects of glucocorticoid excess were first described over 80 years ago, and today glucocorticoid-induced osteoporosis is the most common secondary cause of osteoporosis. Research into the epidemiology, pathophysiology and clinical management of glucocorticoid-induced osteoporosis has produced substantial advances, yet the condition remains under-recognized and under-treated. This review focuses on recent developments in the field and their implications for clinical practice.

## Epidemiology

Continuous oral glucocorticoid therapy is associated with rapid bone loss and an increase in fracture risk that is seen within 3–6 months of initiation and is dose-related. This time course can be explained, at least in part, by the higher doses of glucocorticoids and greater disease activity in the early stages of treatment. Fracture risk remains elevated for the duration of glucocorticoid therapy, but declines after its withdrawal, although whether it reverts to baseline values is unclear [5–8]. Vertebral fractures are particularly characteristic of glucocorticoid-induced osteoporosis, although the risk of non-vertebral fractures, including hip fractures, is also increased.

Recent studies have confirmed the predilection for vertebral fracture, the dose-related increase in fracture risk, and decline in fracture risk with longer duration or discontinuation of glucocorticoid therapy. Amiche et al conducted a Bayesian meta-analysis of fracture risk associated with oral glucocorticoid use, drawing on data from the control groups of clinical trials [9]. In individuals who had initiated glucocorticoid therapy in the last 6 months, the annual incidence of vertebral fracture was 5.1% [95% credible intervals (CrI) 2.8–8.2] and of non-vertebral fracture, 2.5% [95% CrI 1.2–4.2]. For those with duration of glucocorticoid use ≥6 months, the corresponding figures were 3.2% [95% CrI 1.8–5.0] and 3.0% [95% CrI 0.8–5.0]. Using a large administrative database, Balasubramanian et al investigated the effects of initiating systemic (oral or injected) glucocorticoid therapy on fracture risk in patients with new-onset rheumatoid arthritis (mean age 49 years) [10]. Fracture incidence rates were 5 to 9/1000 person years at doses of <15 mg/day, 16 (11, 22.6) at doses ≥15 mg/day and 13.4 (10.7, 16.7) at cumulative doses ≥5400 mg. At 60-182 days after discontinuation of glucocorticoid therapy, fracture risk was 29% lower than in those with ongoing use and by 12 months was similar to non-glucocorticoid users.

The effects of inhaled or intravenous glucocorticoids on fracture risk are less well documented. There is some evidence that high doses of inhaled glucocorticoids may be associated with increased fracture risk, although concurrent use of oral glucocorticoids is often a confounding factor [11–14]. In a large case control study from Denmark, no increase in fracture risk was found in association with other forms of topical steroids [15].

Variation in the severity of adverse effects of glucocorticoids, including bone loss, is well recognized but poorly understood. Pre-receptor modulation of glucocorticoid activity by 11 beta-hydroxysteroid dehydrogenase (11βHSD) enzymes, which interconvert inactive and active cortisone/cortisol, may contribute to this variability through the effects of pro-inflammatory cytokines [16, 17] and genetic polymorphisms in the glucocorticoid receptor gene [18].

## Pathophysiology

### Direct effects on bone

**Fig. 1:**
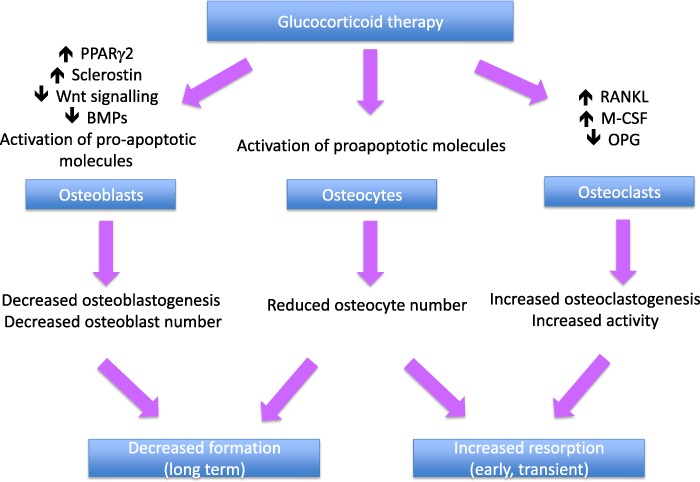
Direct effects of glucocorticoids on bone

Glucocorticoid-induced osteoporosis is characterized by decreased bone formation, with an additional early but transient increase in bone resorption (Fig. 1). The initial increase in remodeling rate is accompanied by reduced bone formation at the level of the individual bone multicellular unit (BMU), and this combination of increased bone turnover and a negative remodeling balance results in rapid bone loss [19–23]. Subsequently, the decrease in bone formation, both at tissue and BMU level, predominates leading to a low turnover state. Direct effects of glucocorticoids on bone formation are mediated largely through upregulation of peroxisome proliferator-activated receptor gamma receptor 2 (PPARγ2) [24] and effects on the Wnt/β-catenin signaling pathway [25, 26]. The former mechanism favors the differentiation of pluripotent precursor cells to adipocytes in preference to osteoblasts, resulting in decreased numbers of osteoblasts. Increased expression of sclerostin, which binds to the co-receptors for frizzled, Lrp4 and Lrp5, results in inhibition of Wnt sigalling leading to reduced differentiation of osteoblast precursors to mature osteoblasts and increased osteoblast and osteocyte apoptosis. The importance of sclerostin in mediating the effects of glucocorticoids on bone formation is emphasized by the demonstration, in mice with sclerostin deficiency, that bone integrity is maintained in the presence of glucocorticoid excess [27]. Furthermore, in a mouse model of glucocorticoid-induced osteoporosis, treatment with an antibody to sclerostin prevented the reduction in bone mass and strength [28].

Glucocorticoids also have direct effects on bone resorption, increasing the production of macrophage colony stimulating factor (M-CSF) and RANKL and decreasing production of osteoprotegerin (OPG) by osteoblastic cells and osteocytes, resulting in an increase both in the number and activity of osteoclasts [29, 30]. This effect diminishes with time, possibly as a result of the reduction in number of osteoblasts and osteocytes. Finally, there is some evidence from animal models that glucocorticoids affect osteocyte morphology and mineralization [31].

### Indirect effects on bone

Other mechanisms that may contribute to glucocorticoid-induced bone loss through indirect effects on bone include hypogonadism, reduced physical activity, increased renal and intestinal losses of calcium, and reduced production of growth hormone, insulin-like growth factor 1 (IGF1) and IGF1 binding protein (IGF-BP) [32]. In addition, the underlying diseases for which glucocorticoid therapy is administered are often associated with increased inflammation, which contributes to bone loss through increased production of pro-inflammatory, pro-resorptive cytokines. Whilst glucocorticoids suppress inflammation and hence should mitigate the adverse effects of inflammation, disease relapse despite therapy is associated with episodes of increased bone resorption. Finally, glucocorticoid excess has adverse effects on muscle mass and function, leading to myopathy and increased risk of falls [33].

### Changes in BMD and bone microarchitecture

Increased rates of bone loss in the hip, spine, and radius are well documented in individuals treated with glucocorticoids. Data obtained from assessment of bone microarchitecture using high-resolution peripheral computed tomography (HRpQCT) are sparse. In a cross-sectional study of 30 postmenopausal women who had received oral glucocorticoids for longer than 3 months, despite similar areal BMD values to 60 control subjects, significantly lower total, cortical and trabecular volumetric BMD, thinner cortices, increased trabecular separation and reduced trabecular number were reported in the radius and tibia; whole bone stiffness, assessed using finite element analysis, was also significantly reduced in comparison to the controls [34]. Although the patients and controls were generally well matched, however, bisphosphonate use was significantly more common in the former (100% vs. 8.6%), so definite attribution of the observed differences to glucocorticoid therapy cannot be made.

Trabecular bone score (TBS) provides an indirect index of trabecular bone architecture that can be obtained from DXA images of the lumbar spine, and has predictive value for fracture independent of BMD [35]. In 64 postmenopausal women who had taken prednisolone in a dose of ≥5 mg daily for >3 months, TBS was significantly lower than in a group of non-glucocorticoid treated controls, although lumbar spine BMD *T*-scores were not significantly different [36]. Similar findings have been reported in 416 individuals on long-term glucocorticoids (≥5 mg daily for 3 months), the decrease in TBS being most marked in men and in individuals with fracture [37]. These findings indicate that glucocorticoids have adverse effects on spine bone microarchitecture that are independent of BMD and which may contribute to increased fracture risk.

### Fracture risk assessment in individuals treated with glucocorticoid-induced osteoporosis

There is evidence from a number of studies that fracture occurs at a higher BMD in individuals receiving glucocorticoids than in non-glucocorticoid treated people [38–40]. This independent effect of glucocorticoid therapy on fracture risk may be due to several factors, including increased falls risk and alterations in bone quality that are not captured by BMD measurements.

Glucocorticoid therapy is included as a risk factor in the FRAX fracture prediction algorithm as a dichotomous variable. “Yes” is entered in the questionnaire if there is current exposure to oral glucocorticoids or past exposure for ≥ 3 months at a dose of 5 mg/day or more of prednisolone or equivalent. Using data from the UK General Research Practice Database, Kanis et al have provided adjustments that can be incorporated into the FRAX calculations to adjust for different doses of glucocorticoids (Table 1) [41]. For daily doses of over 7.5 mg daily of prednisolone or equivalent, greater upward adjustment of fracture probability may be required. It should be noted that the duration of glucocorticoid therapy and cumulative dose are not accommodated within the FRAX algorithm. In addition, the use of total hip BMD in FRAX may result in underestimation of fracture risk in patients with differentially low spine BMD, although a correction for this discordance has been proposed [42, 43]. A final caveat is that the response to treatment in glucocorticoid-treated individuals selected on the basis of FRAX-derived fracture probability has not been documented.

**Table 1:** Adjustment of FRAX-derived fracture probability according to dose of glucocorticoids. Data from ref. [40]

Daily dose of prednisolone (mg)	Average adjustment for major osteoporotic fracture probability	Average adjustment for hip fracture probability
<2.5	−20%	−35%
2.5–7.5	None	None
≥7.5^a^	+15%	+20^a^

^a^For high doses of prednisolone, greater upward adjustment of fracture risk may be appropriate

Assessment of fracture risk using FRAX is recommended in several guidelines for the management of glucocorticoid-induced osteoporosis, including the National Osteoporosis Guideline Group (NOGG) guidance [44, 45], the updated recommendations produced by the American College of Rheumatology (ACR) [46] and guidelines published by the International Osteoporosis Foundation (IOF) and European Calcified Tissues Society (ECTS) [47, 48]. However, FRAX can only be used in people age 40 years and over; in children and young adults, fracture risk assessment should be performed using BMD measurement, together with consideration of other risk factors, particularly previous fracture.

### Management of glucocorticoid-induced osteoporosis

Under-treatment of glucocorticoid-induced osteoporosis is widely recognized [49, 50]. In a population-based study of adults age ≥20 years, rates of BMD testing and prescription of bone protective medication between 1998 and 2008 were studied in individuals prescribed systemic glucocorticoids for 90 days or longer [51]. Overall, in the first six months after initiation of glucocorticoid therapy only 6% had BMD testing, 22% received therapy and 25% had both interventions. Under-treatment was greatest in younger people and men, and primary care physicians had lower prescription rates than rheumatologists. Similar results have been reported more recently using information from a national public health-insurance database in France, with only 8% undergoing BMD testing and prescription of calcium ± vitamin D and bisphosphonates in 18 and 12% respectively [52]. In a large cohort from Canada of men and women aged 66 years or over who were initiating long-term glucocorticoid therapy, Amiche et al reported that only 13% were prescribed bone protective therapy [53]. The problem of under-treatment is compounded by poor persistence with bisphosphonate therapy, particularly in younger people, those with co-morbidities, and those in whom BMD measurements have not been made [54].

### General measures

A number of life-style measures may mitigate the harmful skeletal effects of glucocorticoids, although the evidence base for this approach is weak and requires extrapolation from studies in non-glucocorticoid treated individuals. Falls risk should be assessed and preventive measures instituted when appropriate. Exercise, tailored to the individual patient, and good nutrition with adequate dietary calcium intake should be advocated with avoidance of smoking and alcohol abuse. Maintenance of an adequate vitamin D status should also be advised.

Attention should be paid to keeping the dose of glucocorticoids to a minimum, with the use of steroid-sparing drugs such as methotrexate or azathioprine or alternative routes of administration (for example inhaled or topical) where appropriate. Non-steroidal therapies should be used when possible to maintain remission, once achieved. However, it is also important to maintain suppression of the underlying disease, since this will prevent the adverse skeletal effects of inflammation and other effects of increased disease activity.

Studies of the effects of calcium and/or vitamin D supplementation on BMD in patients taking glucocorticoids have produced conflicting results [55, 56]. However, calcium and vitamin D supplements have been included in most trials of bone protective therapy and should therefore be used as an adjunct to treatment.

### Pharmacological interventions

Regulatory approval of drugs to reduce fracture risk in individuals taking glucocorticoids has been based on the demonstration of similar changes in BMD to those observed in postmenopausal osteoporosis and fracture has been a secondary outcome. For this reason, evidence for anti-fracture efficacy of bone protective agents in glucocorticoid-induced osteoporosis is less robust than that for postmenopausal osteoporosis. In addition, there is inevitable heterogeneity in glucocorticoid-treated trial populations, with respect to age, underlying disease, co-morbidities and co-medications, dose and duration of glucocorticoid therapy and the timing of bone protective therapy. Furthermore, the duration of most treatment studies has been relatively short and this, combined with smaller trial populations, reduces the strength of the safety database.

#### Bisphosphonates

Bisphosphonates are the most commonly used drugs in the management of glucocorticoid-induced osteoporosis. Oral alendronate (5 or 10 mg daily or 70 mg once weekly) and risedronate (5 mg daily or 35 mg once weekly), and intravenous zoledronic acid (5 mg once yearly by intravenous infusion) are all approved for this indication. All have been shown to have beneficial effects on lumbar spine and hip BMD in people treated with glucocorticoids [57–64] and for alendronate and risedronate, there is also evidence from safety or post hoc analyses for a reduction in the rate of vertebral fractures [62, 63]. In the pivotal study of zoledronic acid, a non-inferiority BMD comparison with risedronate, the fracture rate was too low to assess anti-fracture efficacy [64].

The number of non-vertebral and hip fractures has been insufficient in individual trials to assess an impact of bisphosphonates. However, data from cohort studies provide some evidence for efficacy at these sites. In an observational cohort study of women aged >65 years taking alendronate or risedronate, Thomas et al. studied the baseline incidence of clinical fractures in the first three months after starting glucocorticoid therapy and the fracture incidence in the following 12 months [65]. Compared to the baseline incidence, both clinical vertebral and non-vertebral fracture incidence were significantly lower. Using the same database, Overton et al studied the effects of bone protective therapy (bisphosphonates in 95.5%, denosumab or teriparatide in the remaining 4.5%) in a large cohort of new glucocorticoid users [66]. Treatment within the first 90 days of glucocorticoid use was associated with a significant reduction in clinical fractures (including vertebral) of 48% at one year and 32% at three years when compared with non-use. Finally, in three matched cohorts derived from healthcare administrative data from Ontario, Canada, Amiche et al. reported that in individuals initiating long-term glucocorticoids, therapy within the first six months with alendronate or risedronate was associated with a decrease in incident hip fracture (alendronate 0.49 (0.34, 0.69), risedronate 0.58 (0.36, 0.90) [53]. The results confirmed a reduction in vertebral fracture risk with etidronate, alendronate and risedronate, but no decrease in risk of forearm or humerus fractures for any bisphosphonate. The analysis was limited to oral bisphosphonates and zoledronic acid was not considered. Overall, therefore, these studies would be consistent with a beneficial effect of bisphosphonates both on vertebral and non-vertebral fracture, including hip fracture.

The safety profile of bisphosphonates in glucocorticoid-induced osteoporosis has been less well studied than in postmenopausal osteoporosis because of the small number of participants included and shorter duration of the trials. Because of co-morbidities and co-medications, people taking glucocorticoids may be more susceptible to side-effects, for example upper gastrointestinal disorders, and glucocorticoid therapy is a documented risk factor for osteonecrosis of the jaw [67] and probably also for atypical femoral fractures [68, 69]. Bisphosphonates should be used with caution in premenopausal women with child bearing potential, because of the ability of these drugs to cross the placenta.

#### Teriparatide

The predominant role of reduced bone formation in glucocorticoid-induced osteoporosis provides a rationale for the use of anabolic agents in its treatment. In an active-comparator controlled, randomized, double blind study the effects of 18 months treatment with subcutaneous teriparatide, 20 µg/day, or oral alendronate 10 mg/day, were compared in 428 men and women with glucocorticoid-induced osteoporosis [70]. Teriparatide therapy resulted in significantly greater increases in spine and hip BMD and this was seen in both premenopausal and postmenopausal women and in men [71]. The magnitude of increase in BMD was somewhat less than that seen in non-glucocorticoid treated postmenopausal women in another study [72], possibly as a result of the opposing actions of intermittent PTH and glucocorticoids on osteoblastogenesis, and osteoblast and osteocyte apoptosis [73–75].

Although fracture was not a primary end-point of the study, significantly fewer new vertebral fractures occurred in the patients treated with teriparatide when compared to those treated with alendronate (0.6% vs. 6.1%; *p* = 0.004). The incidence of non-vertebral fractures was similar in the two treatment groups. Results after 36 months of treatment demonstrated a continued increase in spine and hip BMD in the teriparatide treated group, with superiority over alendronate at the 24 and 36 month time points [76]. A lower incidence of new vertebral fractures was also seen in the teriparatide group at 36 months (1.7% vs 7.7%, *p* = 0.007), with a similar incidence of non-vertebral fractures in the two groups. Interestingly, measurements of TBS in a subpopulation of this study demonstrated a significant increase after 36 months in teriparatide treated patients, but no significant change in those treated with alendronate [77].

Whilst the long duration of this study is unique amongst treatment trials for glucocorticoid-induced osteoporosis, it should be noted that the participant discontinuation rate at 36 months was 44%. With regard to safety, increased pre-dose serum calcium levels were significantly more common in the teriparatide than alendronate treated group (21% vs. 7%), but no other concerns were identified

In a comparison of the effects of teriparatide and risedronate in men treated with oral glucocorticoids for ≥3 months, significantly greater increases in lumbar spine trabecular volumetric BMD were demonstrated in the teriparatide-treated group at 18 months. In addition, teriparatide was associated with significantly greater improvements in bone strength and stiffness at T12, measured using HRQCT-based finite element analysis [78]. These data, together with the results of the study reviewed above, provide a rationale for the use of teriparatide as a first line option in some patients, particularly those at high risk of vertebral fracture.

Teriparatide is contraindicated in pregnant and lactating women. Women of childbearing potential should use effective methods of contraception while on teriparatide treatment.

#### Denosumab

At present, denosumab is not approved for use in glucocorticoid-induced osteoporosis, although this is currently under consideration by some regulatory agencies. In a Phase 3 randomized double blind active controlled trial of adults taking ≥7.5 mg prednisone or equivalent daily, treatment with denosumab, 60 mg once every 6 months by subcutaneous injection, was associated with a significantly greater increase in lumbar spine BMD compared to risedronate 5 mg daily over a 12 months treatment period [79]. This effect was seen both in patients initiating glucocorticoids and in those established on long-term therapy, regardless of age, race, baseline BMD *T*-score, initial glucocorticoid dose and menopausal status. To date the study has only been reported in abstract form.

### Guidelines for the management of glucocorticoid-induced osteoporosis

National guidelines for the management of glucocorticoid-induced osteoporosis have been produced by a number of countries and updated guidelines have recently been published from the US and UK. Most guidelines address users of long-term (3 months) oral glucocorticoids, and although the daily threshold dose varies it has generally been between 5 and 7.5 mg daily of prednisolone or equivalent. Treatment thresholds also vary; *T*-scores are used in some and generally recognize the higher BMD at which fracture occurs in glucocorticoid-treated individuals. The recent guidelines from the UK and US emphasize the importance of starting bone protective therapy early in high-risk individuals, because of the rapidity with which bone loss and increased fracture risk occur.

The recently updated ACR guideline defines three categories of fracture risk; high, moderate and low. In adults aged ≥40 years, previous osteoporotic fracture, hip or spine BMD *T*-score ≤ −2.5, or 10 year fracture probability of ≥20% (major osteoporotic fracture) or ≥3% (hip fracture) are the criteria for high risk [45]. Moderate and low risk are defined solely on the basis of FRAX-derived fracture probability (10–19% and >1 to ≤3% respectively for moderate risk, <10% and ≤1% respectively for low risk). In adults <40 years old, the criterion for high risk is a previous osteoporotic fracture, whereas moderate and low risk are defined on the basis of BMD. Oral bisphosphonates are recommended as the first line option for individuals at moderate or high risk, with intravenous bisphosphonates, teriparatide, or denosumab in patients who are contraindicated or intolerant to oral bisphosphonates. NOGG, in its 2017 update, takes a similar approach to risk assessment with the use of dose-adjusted FRAX, but uses the UK NOGG intervention thresholds as the basis for making treatment decisions and does not include denosumab as a treatment option [44, 45].

These recent guidelines emphasize the importance of early intervention in high-risk individuals, advocate maintenance of adequate calcium intake and normal vitamin D status, and stress the limited evidence base to support some recommendations, particularly in younger adults. Comparison of the cost-effectiveness of different treatments is problematic because of the lack of fracture data, particularly hip fracture, in clinical trials. The choice of oral bisphosphonates as a first line option in both sets of guidelines is based on their lower cost compared to either zoledronic acid or teriparatide; nevertheless, in view of the superiority of teriparatide vs. alendronate for BMD and vertebral fracture outcomes, it has been suggested that teriparatide should be considered as an alternative first line option in those at highest risk [80].

## Conclusions

There have been significant advances in our understanding of the mechanisms by which glucocorticoids affect bone and increase fracture risk. The clinical management of glucocorticoid-induced osteoporosis, however, remains suboptimal despite the development of more sophisticated approaches to fracture risk assessment and the availability of several effective treatment options. Whilst bisphosphonates are currently the most widely used bone protective drugs in individuals taking glucocorticoids, the use of teriparatide and of denosumab (if approved) as first-line options in some patients merits further investigation. Finally, the potential for sclerostin inhibitors to prevent or reverse adverse skeletal effects of glucocorticoids provides an exciting prospect for future research.
